# “Building palliative care capacity in cancer treatment centres: a participatory action research”

**DOI:** 10.1186/s12904-022-00989-2

**Published:** 2022-06-04

**Authors:** Seema Rajesh Rao, Naveen Salins, Cynthia Ruth Goh, Sushma Bhatnagar

**Affiliations:** 1 Bangalore Hospice Trust – Karunashraya, 40, Varthur Road, Marathahalli, Bengaluru, Karnataka State 560037 India; 2 Department of Palliative Medicine and Supportive Care, Kasturba Medical College, Manipal Academy of Higher Education, Karnataka State, Tiger Circle Road, Madhav Nagar, Udupi District, Manipal, 576104 India; 3 Department of Supportive and Palliative Care, Duke-NUS Medical School, 8 College Road, Singapore, 169857 Singapore; 4 Department of Onco-Anesthesia, Pain and Palliative Care, Institute Rotary Cancer Hospital, All India Institute of Medical Sciences, New Delhi, 110029 India

**Keywords:** Capacity-building, Palliative care, Low-resource setting, Participatory action research

## Abstract

**Introduction:**

There is a significant lack of palliative care access and service delivery in the Indian cancer institutes. In this paper, we describe the development, implementation, and evaluation of a palliative care capacity-building program in Indian cancer institutes.

**Methods:**

Participatory action research method was used to develop, implement and evaluate the outcomes of the palliative care capacity-building program. Participants were healthcare practitioners from various cancer institutes in India. Training and education in palliative care, infrastructure for palliative care provision, and opioid availability were identified as key requisites for capacity-building. Researchers developed interventions towards capacity building, which were modified and further developed after each cycle of the capacity-building program. Qualitative content analysis was used to develop an action plan to build capacity. Descriptive statistics were used to measure the outcomes of the action plan.

**Results:**

Seventy-three healthcare practitioners from 31 cancer treatment centres in India were purposively recruited between 2016 and 2020. The outcome indicators of the project were defined a priori, and were audited by an independent auditor. The three cycles of the program resulted in the development of palliative care services in 23 of the 31 institutes enrolled in the program. Stand-alone palliative care outpatient services were established in all the 23 centres, with the required infrastructure and manpower being provided by the organization. Morphine availability improved and use increased in these centres, which was an indication of improved pain management skills among the participants. The initiation and continuation of education, training, and advocacy activities in 20 centres suggested that healthcare providers continued to remain engaged with the program even after the cessation of their training cycle.

**Conclusion:**

This program illustrates how a transformational change at the organizational and individual level can lead to the development of sustained provision of palliative care services in cancer institutes.

## Introduction

It is estimated that about 2.25 million people are living with cancer in India, with one million new cases every year, and over 0.88 million deaths annually [1]. A majority of them present with advanced metastatic disease, experience moderate-to-severe pain, and require palliative care [2–4]. The modified National Cancer Control Program of India emphasized the need for palliative care at the primary care level [5], and led to the establishment of outpatient pain clinics in cancer centers, government, and private hospitals, stand-alone hospices, outreach clinics, and homecare services [6]. However, the Narcotic Drugs and Psychotropic Substances Act (NDPS) that restricted access to and availability of opioids was a major barrier for pain relief [7]. The NDPS Act was amended in 2014 to make opioids more accessible, but lacked effective implementation [8].

Every year over seven million new patients need palliative care in India, with less than 4% having access to these services [9, 10]. Lack of access to palliative care results in poor symptom control, poor quality of life, inappropriate end-of-life care and increased economic burden [11]. Over 3.5–6.2% of the population in India become poorer every year due to enhanced health expenditure at end of life [12]. Evidence indicates that referral to palliative care results in reduced healthcare spending in patients with cancer and other chronic illnesses [11].

Palliative care activities in India have been on-going for three decades from 1980s. The National Programme for Palliative Care (NPPC) was launched in 2012 [13]. Lack of budget allocation, provider awareness, education and employment opportunities, difficulty in accessing opioids, and absence of legal framework or policies regarding end-of-life-care has impacted timely and effective implementation of the NPPC [14]. A recent country-wide survey of the National Cancer Grid (NCG) [15] centers in India highlighted poor integration of palliative care in oncology [16]. Although India has capacity for generalized palliative care provision as per the Global Atlas of Palliative Care [17], many parts of rural India still have limited access [13]. Bridging the gaps in the capacity to provide palliative care that is cost-effective and equitable necessitates development of these services within the institutions [16]. A national level Cancer Treatment Centers Palliative Care (CTC) program was conceptualized to bridge this gap. In this paper we describe how we used Participatory Action Research (PAR) to design, implement, and evaluate the development of palliative care services in cancer centers in India.

This study aimed to develop palliative care services in cancer centers in India by recognizing receptive organizations and individuals, identifying facilitators and barriers for development of palliative care services, formulating strategies to overcome the constrainers, and creating mechanisms to assess outcomes of program implementation.

## Methods

Participatory action research was used to develop a program to build capacity to provide palliative care in cancer treatment centers in India. In PAR, groups of individuals work together to bring about a change in social or institutional practices [18]. It is a value based, action-oriented, and participatory research [19]. The values of the researcher and the participants inform and drive the research, which leads to an action that brings about the desired change, through a collective process of knowledge generation [20]. Participatory action research proceeds in cyclical stages where the learning of each cycle informs the next [21–23].

The participatory action research was conducted over three cycles from 2015 to 2020 in India. Each cycle had four stages that involved a) developing a critically-informed action plan for social change, b) selecting and implementing the action plan, c) observing the consequences of the actions (evaluation), and d) reflective learning and taking corrective actions. This program was a collaborative partnership between an international palliative care organization and two university teaching hospitals with specialized palliative care units.

Purposive sampling was used to select participants for this program. Medical institutes providing oncology services, both public and private, that were receptive for palliative care, from the states and Union Territories of India where palliative care was underdeveloped or absent were invited to participate in the program [24]. Healthcare practitioners (doctors and nurses) employed in the cancer treatment institutes, willing to take part in the training, ongoing mentoring, and audit were recruited. The three cycles of the CTC program enrolled 54 doctors and 52 nurses from 31 cancer treatment institutes. The physician participants were from the specialties of anesthesia, oncology, internal medicine, psychiatry, and critical care. Nursing participants were from both general and specialty nursing pools.

Multiple sources of data like survey findings, transcripts of focus group discussion, mentor visit notes, and audit findings enabled methodological triangulation and provided rich in-depth information for analysis. Descriptive statistics was used to analyze quantitative data and content analysis to analyze qualitative data. This study was approved by the Institutional Ethics Committee of Kasturba Medical College and Kasturba Hospital, IEC No: 330/2021.

### Developing and implementing the intervention

#### First cycle

##### Plan

A rapid review of literature identified the barriers and enablers to palliative care provision in oncology settings in India [16, 25]. In addition to the challenges posed by population, geographic density and poverty, lack of institutional interest, restrictive opioid policies, poor workforce development, and poor implementation and utilization of available resources for palliative care were identified as major barriers [25]. A core group of national and international palliative care experts participated in a focus group discussion (FGD) to develop a strategy that would enable development of palliative care services in the cancer treatment centers in India [26]. Two experts from the two university teaching hospitals facilitated this FGD. Eight palliative care experts in leadership positions responded to a voluntary call, and consented to be part of the FGD. The findings of the rapid review informed the discussion. This group of experts deliberated on the processes needed to build capacity for palliative care in cancer treatment centers in India. Data from the FGD was collected and analyzed from the moderator notes, recorded conversations, and memory.

The barriers for capacity building identified in FGD included lack of knowledge and skills in palliative care, lack of opioid access, and poor implementation and utilization of available resources. The FGD also deliberated on the strategies for overcoming these barriers. This provided the framework for the CTC palliative care program which was structured focusing on the three components of the WHO Public Health Model [27], education, drug availability and implementation.

Epidemiological and behavioral studies have shown that the critical number of personnel needed to bring about a social or organizational change is 25–30% [28]. When this tipping point is reached, new behaviors are supported and change is inevitable, self-sustaining, and fuels further growth [28]. The FGD deliberated on the critical number needed to bring about this change in India. It was estimated that by establishing palliative care services in at least 100 of the 327 cancer treatment institutes in India, this tipping point would be achievable.

Developing capacity to provide palliative care in an oncology setting requires organizational change as well as individual technical expertise [29]. Organizational climate can either facilitate or impede efforts at capacity building [30]. The FGD deliberated on a bottoms-up approach (individual skill building) and top-down approach (changing agency-specific policies and practices, buy-in from organizational leaders) for sustainable change [31].

Transformative adult learning is enhanced through critical incident analysis, small group discussions, reflective practices, and clinical immersion rotations [32]. This program utilized a workshop model for knowledge development along with problem-based learning thereby enabling improvements in individual knowledge, skills and behavior [33]. This program fostered experiential learning through real-life or simulated scenarios to enhance critical thinking, leadership, team building and collaboration, perspective transformation, and change management competencies [34].

Despite adequate training, the implementation of clinical practices and culture change is a slow and disorganized process and many patients remain deprived of high-quality care that is recommended by the guidelines [35]. Knowledge translation strategies help in addressing this gap. Studies in healthcare sector have highlighted the role of mentorship in improving leadership, management and clinical competencies among the healthcare workers in low-and-middle income countries [36, 37], while being cost-effective, and context-specific [29, 36, 38]. Site-based mentoring was utilized for academic detailing to identify site-specific policies and practices that impede or facilitate organizational change. The FGD also identified the outcome indicators for measuring program implementation. The elements of the FGD are outlined in Table 1.

**Table 1:** Focus group discussion results

Probes	Responses	Recommendations
How can we improve capacity for palliative care within oncology institutes?	Identify change champions for palliative care	• A team of 2 doctors and 2 nurses from each oncology institute to drive the organizational change
	Improve Infrastructure Space, staff, time, equipment	• Stand-alone outpatient palliative care department • Consultation liaison service for inpatients • Task shifting to overcome health workforce shortage • Prevent task overload
	Increase access to opioids	• Procure license to store and dispense opioids • Educate regarding safe practices • Ensure uninterrupted supply
	Initiate advocacy activities to raise awareness about PC	• Develop competency for in-house training • Conduct continuing medical education programs with external support
How can we improve individual capacity to provide palliative care in oncology treatment institutes?	• Improve knowledge and skills about palliative care • Help in knowledge translation	• Attend and complete recommended training in palliative care • Mentoring activities • Academic detailing • Develop institutional policies and guidelines
What are the outcome indicators to measure the implementation of the program?	For organizational capacity building	• Number of dedicated staff for palliative care • Number of hours of OPD per month • Number of patients seen in OPD per month • Number of patients seen in CL per month • Number of new patients referred to palliative care
	For individual capacity building	• 5-day face-to-face training in Palliative Care • 5-day clinical attachment at specialist palliative care institute • 2-day mentorship training by a visiting mentor • Completion of certificate course in essentials of palliative care (CCEPC) by Indian Association of Palliative Care
	For morphine availability	• Number of milligrams of morphine use per month
	For advocacy activities	• Number of trainings conducted in a year • Number of doctors trained • Number of nurses trained • Number of allied healthcare practitioners trained • Observance of World Hospice and Palliative Care Day in the institute

The first cycle was initiated in January 2016. Thirty healthcare practitioners (16 nurses and 14 doctors) from 10 cancer treatment institutes participated in the training program. The duration of the first cycle was 24 months.

##### Act

In this stage all 30 participants were provided training in palliative care. All participants underwent a 5-day residential face-to-face training program along with a 5-day clinical attachment at a specialist palliative care institute. A senior palliative care expert mentored the palliative care activities at each participating institute. This stage took four months. Each team on completion of the training initiated palliative care services within their respective institute. Organizational restructuring and task-shifting helped institutes overcome the workforce shortage in palliative care. Physical space for an outpatient department was identified and established. Healthcare personnel trained in the CTC program staffed the palliative care OPD, initially part-time. Consultation liaison services were established for inpatient care. Engaging with senior healthcare leadership and opinion leaders for improving access to opioids and ensuring uninterrupted supply was one of the key focuses of the action plan. In addition, the local change champions organized training activities with external resource personnel for all cadres of healthcare personnel within their institutes, thereby increasing the workforce available for palliative care. The action plan also involved building partnerships by engaging with community and local leaders to advocate for palliative care. This process took about 12 months.

##### Observation and evaluation

Both qualitative and quantitative methods were used to evaluate the action plan, longitudinally at different stages of the cycle. Pre- and post-evaluation questionnaires; survey forms that explored institutional activities like current care practices, organizational culture, barriers and enablers; mentor visit notes; and audit findings were used for evaluation. At the end of each cycle, an external auditor evaluated the implementation of project objectives. Of the 10 cancer treatment institutes enrolled in the CTC program, five centers were able to develop capacity to provide palliative care.

##### Reflections from the first cycle

The evaluation results of the first cycle were shared with the participants and faculty. The first cycle was followed by group reflective sessions. Rolfe’s reflective model was used to better understand the barriers and enablers and modify the action plan for the second cycle accordingly [39]. This process took about six months. Literature has shown that local change champions are crucial in supporting transformative change efforts within an organization [33, 36]. Senior faculty in leadership roles were enrolled into the training program. A 3-day face-to-face centralized refresher course was incorporated into the regular training. The focus on small group problem-based learning (PBL) and peer learning during the refresher course enhanced collaboration and communication within and between members of the group [40]. More structured mentorship visits were initiated.

The barriers and adaptations are outlined in Table 2.

**Table 2:** Reflections from the first cycle of the CTC Program

Organizational Barriers	
Lack of buy-in from administrators and decision-makers	• A more rigorous process of sample selection through interviews and personal judgement • Identifying senior clinical and non-clinical leaders within organizations and engaging them
Workforce shortage to initiate PC service	• A 2-day on-site structured mentor visit where mentors addressed site-specific issues with administrators/decision-makers • Sensitization and training programs by experts on palliative care
Lack of resources – space, funding, time	
Lack of awareness about PC among other healthcare providers	
Hierarchical structure in the healthcare system that impedes communication and collaboration	• Group brainstorming with the team on how to enhance team collaboration and communication • Team building activities during the mentor visit and refresher course
Individual Barriers:	
Lack of motivation towards PC	• Improving selection of change champions by interviews and personal judgment • Selecting those with some awareness, understanding and commitment towards palliative care • Identifying those who have had short-term training in palliative care in the cancer treatment institutes and involving them in the program
• Deficits in PC skills and knowledge • Lack of leadership skills	• Structured training program that included a 3-day centralized refresher training which focused on: • Problem-based learning and peer learning techniques to foster a culture of continuous self-directed learning • Microlearning to reinforce previously acquired knowledge and skills, address gaps in knowledge and help in retention
Competing interests of the healthcare provider	• Appropriate selection of candidates who would be able to devote exclusive time to PC • Organizational buy-in from administrators ensured to smoothen this transition
Barriers for drug availability	
Opioid access and use	• Liaising with opinion leaders, administrators, local change champions, and governmental agencies for better opioid access • Training for healthcare staff on safe use of opioids

#### Second cycle

The second cycle was initiated in January 2018, with nine cancer treatment institutes and 32 participants; 16 doctors and 16 nurses. The duration of this cycle was 12 months. Palliative care services were established in eight of the nine centers.

#### Third cycle

The third cycle was initiated in January 2019, with 12 cancer treatment institutes enrolled in this cycle, with 44 participants; 22 doctors and 22 nurses. The duration of this cycle was 12 months. The growing number of participants and centers needed more continuous engagement of change champions. A full-time PC consultant was appointed to engage and monitor the program activities. Palliative care services were successfully established in 10 of the 12 institutes that had participated in the training.

**Fig. 1:**
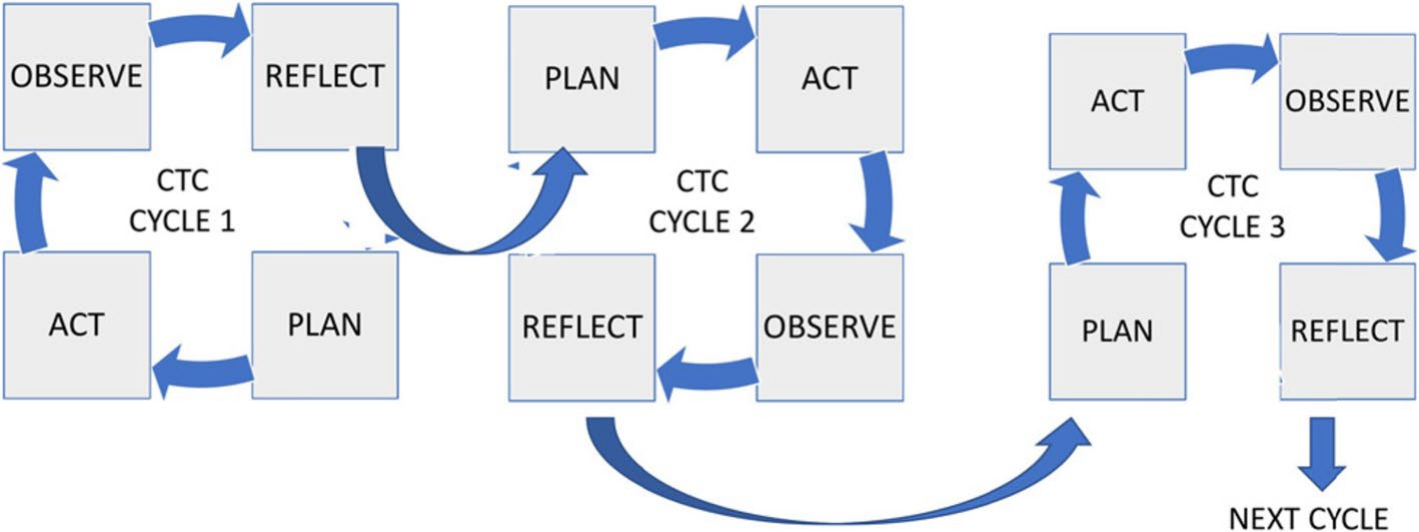
Participatory action research cycle of the cancer treatment centers program

The three cycles and steps of the research process are depicted in Fig. 1.

## Results

The overall impact of the interventions of CTC program is presented in the Table 3.

**Table 3:** Overview of the CTC program

Sl No	CTC Program	Year	Number of institutes enrolled	Number of centers who completed training	Number of HCP trained in palliative care
**1**	First cycle	2016–2018	10	5	13
**2**	Second cycle	2018–2019	9	8	27
**3**	Third cycle	2019–2020	12	10	33
	**Total**		**31**	**23**	**73**

**Fig. 2:**
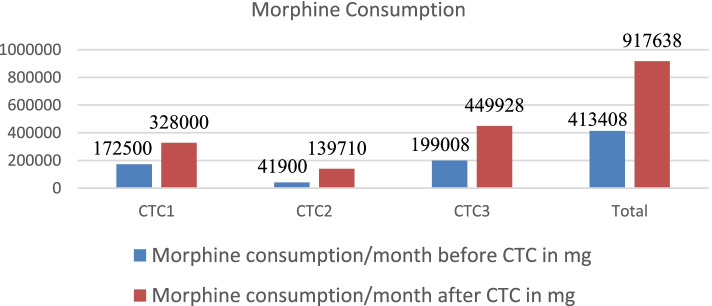
Comparison on morphine consumption in CTC centers

Outcome Indicators: The outcome of the CTC programs was measured by the indicators that were defined a priori and included development of infrastructure (OPD space, personnel, and time), drug availability and training. These are listed below:

- Establishment of Palliative care outpatient services in the cancer treatment institutes: While 12 cancer treatment institutes had independent palliative care outpatient departments before CTC program, the number increased to 23 after CTC training. The number of healthcare providers working either part-time or full-time increased from 36 to 174 after initiation of the program.
- Morphine availability and use: While 14 institutes had the license to procure, store, and dispense morphine before the program, it increased to 23 after the CTC program. Total morphine consumption per month in the cancer treatment institutes increased from 413,408 mg to 917,638 mg, indicating increased availability and use. The use of morphine is depicted in Fig. 2.
- Training and Advocacy Activities: None of the centers were engaged in palliative care training or advocacy activities before CTC program. After CTC training 20 of the centers have initiated both in-house training and advocacy activities.
- Secondary Outcomes: There were several meaningful outcomes of this program, which were not quantifiable. Palliative care services were established in medical colleges in states and Union Territories of India which hitherto had no or minimal access to palliative care. The change champions have assumed leadership roles in developing palliative care policies and have initiated postgraduate training in palliative care.

## Discussion

Relief from pain and serious health-related suffering is a human right [24]. The disparity between need and availability of palliative care continues to persist, especially in lower and middle-income countries (LMIC) [9]. The Quality of Death Index published by the Economist Intelligence Unit in 2015 that measured the quality of palliative care around the world, ranked India 67^th^ out of 80 countries in palliative care provision [25]. The barriers to development of palliative care in the oncology setting are complex and traverse multiple domains including policy, education, drug availability, awareness, advocacy, and implementation.

The central aim of PAR is to bring about a social change where groups of individuals work together collaboratively to bring about the change [19]. PAR fosters capacity development in all those who participate in the process. The CTC Program was envisaged to bring about a transformational change in practice in the cancer treatment institutes in India through capacity building for palliative care. This program was able to help 23 cancer treatment institutes build capacity to provide palliative care. The PAR framework aided the development of action plan that adopted a) appropriate context-specific capacity-building strategies [41], b) adult transformative learning for knowledge-sharing and knowledge-translation [34, 42], and c) partnerships between organizations [31].

Research shows that when changes are planned and executed by those within the organization then capacity building is sustainable and successful [31]. The participants in the CTC program were the drivers of change, who identified with the common goal of developing capacity to provide palliative care within their institutions, and developed an action plan along with the researchers to achieve this goal. The participants and researchers revisited, evaluated, and redefined the outcomes with each cycle. This reflective cycle enabled identification of components that facilitated and impeded palliative care capacity building in the cancer treatment institutes and facilitated corrective actions for the next cycle [39].

In the four-tier hierarchy of capacity building needs, this model focused on the individual and institutional level needs, that is, staff and facilities, skills and tools [41]. To initiate, develop and implement any change you need change champions, both individual and organizational [43]. Identifying and training the change champions was the first step. The champions through advocacy activities in the local, national, and regional level have enhanced palliative care service provision in many states and union territories. Our experience suggests that while the participating institutes were able to bring about a transformative change and establish palliative care services within their institution, they were able to facilitate changes beyond their institution. Some of the participating centers were able to enlist and train other institutions. This dependence on change champions was also responsible for the failure to capacity build in some institutes. When the change champions lacked skills in leadership and teamwork, they were ineffective in starting the services within their institutes. When the change leaders resigned from the institute, the established services collapsed. Developing a structured selection process to identify organizational and individual champions will help in preventing dropouts. Strengthening the fourth pillar of capacity-building, that is, structures, systems and roles will help in overcoming this weakness in the program.

Education and training are important components of capacity building as is knowledge translation [42]. Adult transformative learning strategies were applied in this program [34]. The structured residential training program was as per national standards and aided conceptual learning. The refresher course created the space for the newly formed palliative care teams to share their experiences and aided experiential and peer-assisted learning. The rapid feedback through quizzes enabled participant learning and knowledge durability. It is important for knowledge learned to be translated into practice, that is, the knowledge to action cycle needs to be completed. The peer-based mentorship model helped the individual champions in implementing the learning in their institutes [40].

Partnerships were integral for the implementation of this project. Partnerships with international and national organizations were crucial for this project. The international organization provided resources for the training and mentoring activities. The aim of this initiative was to ensure sustainability of this process. The PAR approach co-opted the participants as partners who brought about a change in the institution’s culture and attitude towards palliative care. The buy-in by the institutional and administrative leaders ensured sustainability of the program in the 23 centers without external support. In addition, collaborative partnerships between individual team members and between other teams ensured sustainability. Doctors and nurses from individual institutes worked together for the first time as a team during the CTC training and developed a team and group identity with a shared mission and vision. The interactive environment of the residential face-to-face training aided collaboration, communication and networking between individuals and institutes fostering partnerships.

The experience with the three CTC cycles also showed that what was transformative and worked in one setting is not necessarily relevant and applicable in the other. For the process to be successful more attention needs to be paid to the contextual starting conditions, facilitation skills of the mentors, and the leadership skills of the change champions. Lack of a structured process of selection, training and evaluation is one of the limitations of the program. A more robust training program incorporating a flipped classroom model, and standardized reading resources and training evaluation metrics is being planned for the subsequent cycles. The CTC program has primarily focused on personal and performance capacity without focusing on the structural and system capacity. This is another limitation of the program. The development of the program did not factor in the end user, the patient and the family experiences, and is another major limitation of this program. These would need to be incorporated in the next cycle of the program.

There were methodological limitations in the study. The purposive sampling could have led to selection bias, choosing those centers where programs were likely to succeed. The data collection was achieved through pre-existing self-reported data, survey questionnaires, field notes, and through interviews which could have led to response and researcher bias. However, these were mitigated through methodological and data triangulation.

### Directions for future research

The purpose of the CTC program is to increase the capacity to provide high quality palliative care to patients with terminal illnesses. A service evaluation of the project against national standards with validated evaluation tools is planned. Exploring patient and caregiver experience with the focus on symptom control, quality of life, and patient and family satisfaction will help us to define the effectiveness of the project. In addition, we plan to conduct a qualitative study to explore the participating healthcare providers’ views of the components of the CTC program, and how it impacted development of PC services in their institutes, positively and negatively. Participants from both the successful and unsuccessful centers will be included in this study. We hope to incorporate the learnings from these studies to improve the model of CTC program in subsequent cycles.

## Conclusion

Palliative care needs to be incorporated into the routine care of patients with cancer and other terminal illnesses. The challenges in implementing this can be mitigated if we develop a sustainable working model to build capacity to provide palliative care. The CTC program focused on capacity building, knowledge development and translation and partnership between national and international palliative care organizations, individual and organizational stakeholders, and change champions. This program aimed at bringing about a transformational change at the organizational as well as at an individual level, led to development of palliative care services in cancer treatment institutes in India. Developing the institution-based generalist palliative care model in cancer institutes might enhance capacity to provide palliative care in India and might bridge the healthcare inequities related to palliative care access.

## Data Availability

The datasets used and analysed for this study can be accessed through the Kasturba Medical College Research Cell. It is available from the corresponding author on reasonable request at the following email: sushmabhatnagar1@gmail.com.
